# Action Tremor Asymmetry Profile Does Not Aggregate in Families with Essential Tremor

**DOI:** 10.3389/fneur.2017.00148

**Published:** 2017-04-19

**Authors:** Elan D. Louis, Nora Hernandez, Karen P. Chen, Kelly V. Naranjo, Jemin Park, Lorraine N. Clark, Ruth Ottman

**Affiliations:** ^1^Division of Movement Disorders, Department of Neurology, Yale School of Medicine, Yale University, New Haven, CT, USA; ^2^Department of Chronic Disease Epidemiology, Yale School of Public Health, Yale University, New Haven, CT, USA; ^3^Center for Neuroepidemiology and Clinical Neurological Research, Yale School of Medicine, Yale University, New Haven, CT, USA; ^4^College of Physicians and Surgeons, Taub Institute for Research on Alzheimer’s Disease and the Aging Brain, Columbia University, New York, NY, USA; ^5^Department of Pathology and Cell Biology, Columbia University Medical Center, New York, NY, USA; ^6^G.H. Sergievsky Center, College of Physicians and Surgeons, Columbia University, New York, NY, USA; ^7^Department of Neurology, College of Physicians and Surgeons, Columbia University, New York, NY, USA; ^8^Department of Epidemiology, Mailman School of Public Health, Columbia University, New York, NY, USA; ^9^Division of Epidemiology, New York State Psychiatric Institute, New York, NY, USA

**Keywords:** essential tremor, movement disorders, genetics, familial aggregation, clinical

## Abstract

**Background:**

Action tremor is the hallmark feature of essential tremor (ET). While the tremor typically is mildly asymmetric, in some patients, it is markedly asymmetric. There are few data on factors that influence this asymmetry. ET is also a highly familial disease. Whether the tremor asymmetry profile (i.e., differential expression of tremor in each arm in a given patient) is similar across family members is not known. The alternative possibility is that this feature is not heritable. There are no published data addressing this issue. The aim of this study was to determine whether the extent of action tremor asymmetry ran in ET families.

**Methods:**

ET probands and relatives were enrolled in a genetic study at Yale and Columbia Universities. An in-person evaluation included a videotaped neurological examination, including a detailed assessment of tremors. A senior movement disorders neurologist reviewed all videotaped examinations, and the severity of postural and kinetic arm tremors was rated on 12 examination items using a reliable rating scale. The tremor asymmetry index = right arm tremor score − left arm tremor score. We used a bivariate linear regression model to assess the predictors of the tremor asymmetry index in relatives; this model used the tremor asymmetry index in the proband as a primary predictor of interest. In an analysis of variance (ANOVA), we tested for heterogeneity across families in the tremor asymmetry index (i.e., to see whether there was a significant family effect).

**Results:**

There were 187 enrollees (59 probands, 128 affected relatives). In a bivariate linear regression model, the tremor asymmetry index in the proband was not a predictor of the tremor asymmetry index in their relatives (*p* = 0.66). In an ANOVA, family grouping did not explain a significant proportion of the total variance in the tremor asymmetry index (*p* = 0.56).

**Conclusion:**

Tremor asymmetry did not aggregate in families with ET. Therefore, this does not seem to be a disease feature that is heritable. These data will provide added value to the clinical dialog, giving patients one more piece of information about the way the disease manifests within families.

## Introduction

In general, neurodegenerative disorders do not involve both sides of the brain to an equal degree and, as a result, associated motor features are often asymmetric. For example, the motor features of Parkinson’s disease (PD) typically involve one side of the body more than the other (1–3).

Essential tremor (ET) is one of the most prevalent neurological diseases (4–6). Typically, action tremor, which is the hallmark feature of ET, is mildly asymmetric. In one study of ET patients, clinical ratings of action tremor were assigned to each arm; on average, there was a 1.32-fold difference between sides and, as further measured by quantitative computerized tremor analysis, a 1.71-fold mean difference between tremor amplitudes in each arm (7). In some patients, the tremor can be very asymmetric and, according to some estimates, in 4% of patients the tremor is unilateral (8). A possible cause of the motor asymmetry in diseases such as ET and PD is differential involvement of bilateral brain structures due to selective vulnerability (1, 9).

Essential tremor is a highly familial disorder (10–12). Treating physicians often care for patients who have multiple affected family members and other at-risk family members. Whether motor asymmetry is heritable, and is passed from one family member to another, is not known. Several clinical features of ET run in families [e.g., age of tremor onset (13) and rate of tremor progression (14)], whereas others do not [e.g., presence of cranial tremor (15)]. Whether the tremor asymmetry profile (i.e., differential expression of tremor in each arm) is similar across family members is not known. The alternative possibility is that this feature is not heritable. At present, the state of the field is that there are no published data addressing this issue and, hence, there is a gap in knowledge. Such data would be useful to treating physicians in providing additional prognostic framework and family guidance information to their patients and families who suffer from this disease.

Essential tremor cases (probands) and their relatives were enrolled in a genetic study. We tested the specific hypothesis that action tremor asymmetry would run in ET families.

## Materials and Methods

### Ascertainment of Probands

Essential tremor cases (probands) and their reportedly affected relatives were enrolled in a study of ET, the Family Study of Essential Tremor (FASET) (2011–present) (14). The study was advertised on several ET society websites. The three inclusion criteria for probands were (1) a diagnosis of ET had been assigned by a doctor, (2) age of tremor onset ≤40 years (later changed to ≤50 to be more inclusive), (3) ≥2 living relatives in the United States who have ET that was diagnosed by a doctor; these relatives were not reported to have dystonia or PD. The exclusion criterion for probands was a prior diagnosis of dystonia or PD. Potential ET probands contacted the FASET study coordinator. Prior to final selection for enrollment, a set of four Archimedes spirals (two right, two left) were submitted by probands and rated by a senior neurologist specializing in movement disorders (Elan D. Louis). Probands were included if one or more of the spirals had a Washington Heights Inwood Genetic Study of Essential Tremor rating that indicated moderate or greater tremor (16).

### Ascertainment of Relatives

Based upon a telephone interview with the proband, relatives with ET were identified (14). With the proband’s permission, these relatives were then contacted by telephone and were pre-enrolled if they reported the presence of tremor in the absence of a prior diagnosis of dystonia or PD. Prior to final selection for enrollment, four Archimedes spirals were submitted by relatives and rated by Elan D. Louis. Relatives were included if one or more of the spirals indicated moderate or greater tremor (16).

### In-Person Evaluation

An in-person evaluation was then conducted in enrollees’ homes; this included several questionnaires (e.g., demographic features, tremor features, medical history, and medications) and a videotaped neurological examination (14). The latter included a detailed assessment of postural, kinetic, intention, and rest tremors, as well as dystonia and other movement disorders (17). Elan D. Louis reviewed all videotaped examinations, and the severity of postural and kinetic arm tremors was rated on 12 examination items using a reliable rating scale (18). As reviewed elsewhere (19, 20), ratings were 0, 0.5, 1.0, 1.5, 2, 3, and 4 and resulted in a tremor score in the right arm [range = 0–23 (maximum)], a tremor score in the left arm [range = 0–23 (maximum)], and a total tremor score [range = 0–46 (maximum)] (17). The tremor asymmetry index was the tremor score in the right arm − the tremor score in the left arm. In addition, cases were classified into three categories: (1) higher tremor score on right than left, (2) higher tremor score on left than right, and (3) tremor scores on both sides that were equal.

### Diagnoses

All ET diagnoses in probands and relatives were reconfirmed based on review of questionnaires and videotaped neurological examination data. Diagnoses of ET in probands and relatives were assigned based on published diagnostic criteria [moderate or greater amplitude kinetic tremor during three or more activities or a head tremor in the absence of PD or another known cause (e.g., medication-induced tremor and tremor from hyperthyroidism)] (14, 16, 18).

### Final Sample

There were 274 enrollees. We excluded 50 enrollees who came from families in which either the proband had not yet been enrolled or at least one relative had not yet been enrolled. We also excluded nine enrollees who had had surgery for ET (seven deep brain stimulation and two thalamotomy). We also excluded the relatives of these nine probands.

The final sample (187 enrollees) included 59 probands and 128 affected relatives (105 first-degree, 16 second-degree, and 7 third-degree).

### Statistical Analyses

Analyses were performed in SPSS (Version 21.0). Probands’ vs. relatives’ characteristics were compared using Student’s *t*-tests, chi-square tests, and Fisher’s exact tests (Table 1). We also assessed the clinical correlates of the tremor asymmetry index using Student’s *t*-tests, analysis of variance (ANOVA), and Pearson’s correlation coefficients (Table 2).

**Table 1:** **Demographic and clinical characteristics of 187 cases**.

	Probands (*N* = 59)	Affected relatives (*N* = 128)	Significance
Age (years)	64.1 ± 15.0, 22–91	60.5 ± 17.2, 20–93	*p* = 0.18^a^
Female gender	38 (64.4)	64 (50.0)	*p* = 0.07^b^
White race	55 (93.2)	121 (94.5)	*p* = 0.74^c^
Right-handed	57 (96.6)	117 (91.4)	*p* = 0.23^c^
Relationship to proband			NA
Self	59 (100)	0 (0.0)	
Child	0 (0.0)	33 (25.8)	
Sibling	0 (0.0)	57 (44.5)	
Parent	0 (0.0)	15 (11.7)	
Grandchild	0 (0.0)	3 (2.3)	
Aunt/uncle	0 (0.0)	4 (3.1)	
Nephew/niece	0 (0.0)	9 (7.0)	
Other (third degree)	0 (0.0)	7 (5.5)	
Total tremor score (neurological examination)	23.5 ± 5.1, 12.5–35.5	18.7 ± 5.0, 8.0–32.0	*p* < 0.001^a^
Tremor score in right arm (neurological examination)	11.5 ± 3.1, 2.5–20.0	9.1 ± 2.8, 1.5–17.0	*p* < 0.001^a^
Tremor score in left arm (neurological examination)	11.9 ± 2.5, 7.0–17.5	9.5 ± 2.2, 4.5–18.0	*p* < 0.001^a^
Tremor asymmetry index = tremor score in right arm − tremor score in left arm (neurological examination)	−0.4 ± 2.5	−0.4 ± 2.2	*p* = 0.90^a^
Side in which tremor score is higher			*p* = 0.07^b^
Right	19 (32.2)	43 (33.6)	
Left	28 (47.5)	74 (57.8)	
Equal	12 (20.3)	11 (8.6)	
Currently takes daily medication for essential tremor	38 (64.4)	33 (25.8)	*p* < 0.001^b^
Age of tremor onset (years)	22.4 ± 14.8	30.9 ± 19.2	*p* = 0.001^a^
Duration of tremor (years)	41.7 ± 18.3	30.2 ± 17.9	*p* < 0.001^a^

*All values are mean ± SD, range, or number (%), unless otherwise specified*.

*NA, not applicable*.

*^a^Student’s t-test*.

*^b^Chi-square test*.

*^c^Fisher’s exact test*.

**Table 2:** **Clinical correlates of the tremor asymmetry index in 187 essential tremor (ET) cases**.

	Probands (*n* = 59)	Relatives (*n* = 128)
Age (years)	*r* = 0.15, *p* = 0.25	*r* = 0.08, *p* = 0.36
Gender		
Male	−0.6 ± 2.4	−0.3 ± 2.2
Female	−0.3 ± 2.5	−0.4 ± 2.4
	*p* = 0.63^a^	*p* = 0.92^a^
Race		
White	−0.3 ± 2.2	−0.4 ± 2.1
Other	−1.5 ± 5.6	0.0 ± 3.0
	*p* = 0.71^a^	*p* = 0.65^a^
Handedness		
Right	−0.4 ± 2.5	−0.4 ± 2.2
Left	0.0 ± 1.4	0.1 ± 1.7
	*p* = 0.82^a^	*p* = 0.47^a^
Relationship to proband		
Self	−0.4 ± 2.5	
Child		−0.8 ± 1.8
Sibling		−0.4 ± 2.0
Parent		0.5 ± 2.5
Grandchild		−2.2 ± 1.6
Aunt/uncle		1.6 ± 5.4
Nephew/niece		−0.6 ± 2.3
Other (third degree)		0.1 ± 1.7
		*p* = 0.14^b^
Total tremor score (neurological examination)	*r* = 0.27, *p* = 0.04	*r* = 0.29, *p* = 0.001
Tremor score in right arm (neurological examination)	*r* = 0.61, *p* < 0.001	*r* = 0.62, *p* < 0.001
Tremor score in left arm (neurological examination)	*r* = −0.22, *p* = 0.09	*r* = −0.19, *p* = 0.03
Side in which tremor score is higher		
Right	2.0 ± 1.5	1.9 ± 1.7
Left	−2.2 ± 2.0	−1.8 ± 1.2
Equal	0.0 ± 0.0	0.0 ± 0.0
	*p* < 0.001^b^	*p* < 0.001^b^
Currently takes daily medication for ET		
Yes	−0.01 ± 2.6	−0.6 ± 2.3
No	−1.1 ± 2.0	−0.3 ± 2.2
	*p* = 0.10^a^	*p* = 0.41^a^
Age of tremor onset (years)	*r* = 0.04, *p* = 0.78	*r* = −0.007, *p* = 0.94
Duration of tremor (years)	*r* = 0.10, *p* = 0.47	*r* = 0.03, *p* = 0.76

*The table demonstrates the correlation between the tremor asymmetry index and clinical variables (e.g., age and age of tremor onset) or the value of the tremor asymmetry index (mean ± SD) across categories of clinical variables (e.g., males vs. females, whites vs. others)*.

*r, Pearson’s r*.

*^a^Student’s t-test*.

*^b^Analysis of variance*.

We used a bivariate linear regression model to assess the predictors of the tremor asymmetry index in relatives; this model used the tremor asymmetry index in the proband as a primary predictor of interest. In these models, assumptions of linearity, independence, homoscedasticity, and normality were all met. Because of the non-independence of proband–relative pairs within each family, for this model, we used generalized estimating equations (GEEs) to compute beta and *p* values. In additional GEE analyses, we also stratified our sample into first-degree vs. second-degree relatives vs. third-degree relatives and by genetic load (i.e., number of enrolled affected relatives). In multivariate linear regression models using GEE, other predictors that we considered included the relative’s age, gender, race, relationship to the proband, daily use of medication for ET, age of tremor onset, duration of tremor, and total tremor score.

We performed several additional analyses. First, we selected subjects whose tremor asymmetry index had extreme values (the top 10% of subjects whose tremor asymmetry index value was ≤−2.5 and the bottom 10% of subjects whose tremor asymmetry index value was ≥2.5), and in a bivariate linear regression model (GEE) assessed whether the tremor asymmetry index in the proband was a predictor of the tremor asymmetry index in the relatives. Second, we selected probands whose tremor asymmetry index had extreme values, and in a bivariate linear regression model (GEE) assessed whether the tremor asymmetry index in the proband was a predictor of the tremor asymmetry index in the relatives. Third, cases were classified into three categories: (1) higher tremor score on right than left, (2) higher tremor score on left than right, and (3) tremor scores on both sides that were equal. We used a bivariate linear regression model to assess whether this classification of tremor asymmetry in the proband predicted this classification of tremor asymmetry in the relatives. Finally, in an ANOVA, we tested for heterogeneity across families in the tremor asymmetry index (i.e., to see whether there was a significant family effect); in this model, the dependent variable was the tremor asymmetry index in the relatives and the group factor was the family number.

## Results

The characteristics of enrollees are shown (Table 1); probands differed from their affected relatives in a number of respects (tremor severity on examination, use of tremor medications, age of tremor onset, duration of tremor) and marginally in other respects (gender). While the tremor asymmetry index was nearly identical in the two groups, there was a marginal difference in terms of the side in which tremor was more severe (Table 1). Of 59 probands, 15 (25.4%) had at least 1 other enrolled affected relative, 28 (47.5%) had 2, 8 (23.6%) had 3, and 8 (23.6%) had 4 or more.

We examined the clinical correlates of the asymmetry index (Table 2). It was not related to age, gender, race, relationship to the proband (among relatives), daily use of medication for ET, age of tremor onset, or duration of tremor. It was associated with tremor scores (Table 2).

**Figure 1:**
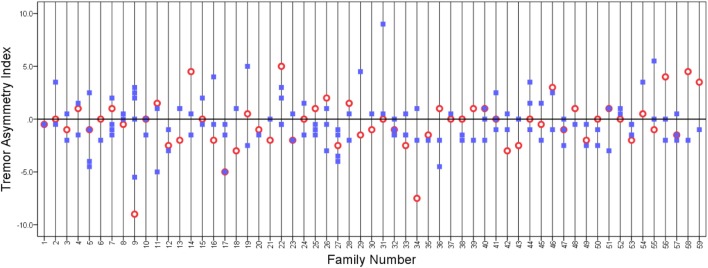
**Tremor asymmetry index in probands (open circles) and relatives (closed squares)**. A value of 0 indicates that the tremor was equal on both sides. Positive values indicate that tremor is greater on the right side, and negative values indicate that tremor is greater on the left side. Vertical grid lines run through the data points in each family.

We plotted the tremor asymmetry index in probands and their relatives (Figure 1). There seemed to be no identifiable pattern.

In a bivariate linear regression model, the tremor asymmetry index in the proband was not a predictor of the tremor asymmetry index in the relatives (beta = 0.029, *p* = 0.66). We then stratified our sample into first-degree, second-degree, and third-degree relatives. In bivariate linear regression models, the tremor asymmetry index in the proband was not a predictor of the tremor asymmetry index in first-degree relatives (beta = 0.003, *p* = 0.98), in second-degree relatives (beta = 0.08, *p* = 0.30), or in third-degree relatives (beta = 0.56, *p* = 0.12). We then stratified our sample by genetic load (i.e., number of enrolled affected relatives); in strata of increasing load, there was no increase in the association between tremor asymmetry index in the proband and tremor asymmetry index in the relatives.

In a series of multivariate linear regression models, other predictors that we considered, one by one, included the relative’s age, gender, race, relationship to the proband, daily use of medication for ET, age of tremor onset, duration of tremor, and total tremor score. Aside from the total tremor score, which was associated with the tremor asymmetry index in the relatives (beta = 0.14, *p* = 0.001), none of these variables was associated with the tremor asymmetry index in the relatives when it was included in a two-variable model along with the tremor asymmetry index in the proband (all *p* values >0.05); in each model there was similarly no association between the tremor asymmetry index in the relatives and the probands (all *p* values >0.05).

**Figure 2:**
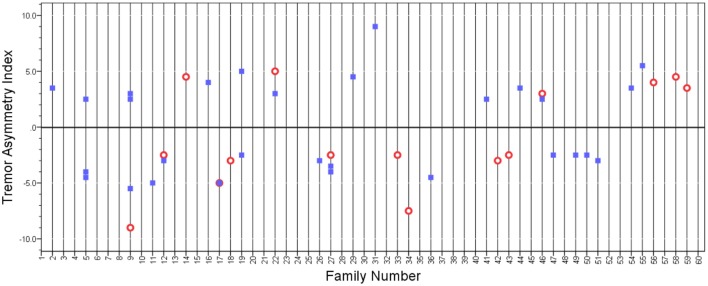
**Tremor asymmetry index in probands (open circles) and relatives (closed squares) whose tremor asymmetry index had extreme values (i.e., value was above or below a certain threshold)**. A value of 0 indicates that the tremor was equal on both sides. Positive values indicate that tremor is greater on the right side, and negative values indicate that tremor is greater on the left side. Vertical grid lines run through the data points in each family.

We performed several additional analyses. First, we selected subjects whose tremor asymmetry index had extreme values. These were the top 10% of subjects whose tremor asymmetry index value was ≤−2.5 and the bottom 10% of subjects whose tremor asymmetry index value was ≥2.5. There were 44 such subjects, including 15 probands and 29 relatives. There seemed to be no patterning of the relatives’ asymmetry index based on that of the probands’ (Figure 2) and in the bivariate linear regression model, the tremor asymmetry index in the proband was not a predictor of the tremor asymmetry index in the relatives (beta = 0.16, *p* = 0.30).

**Figure 3:**
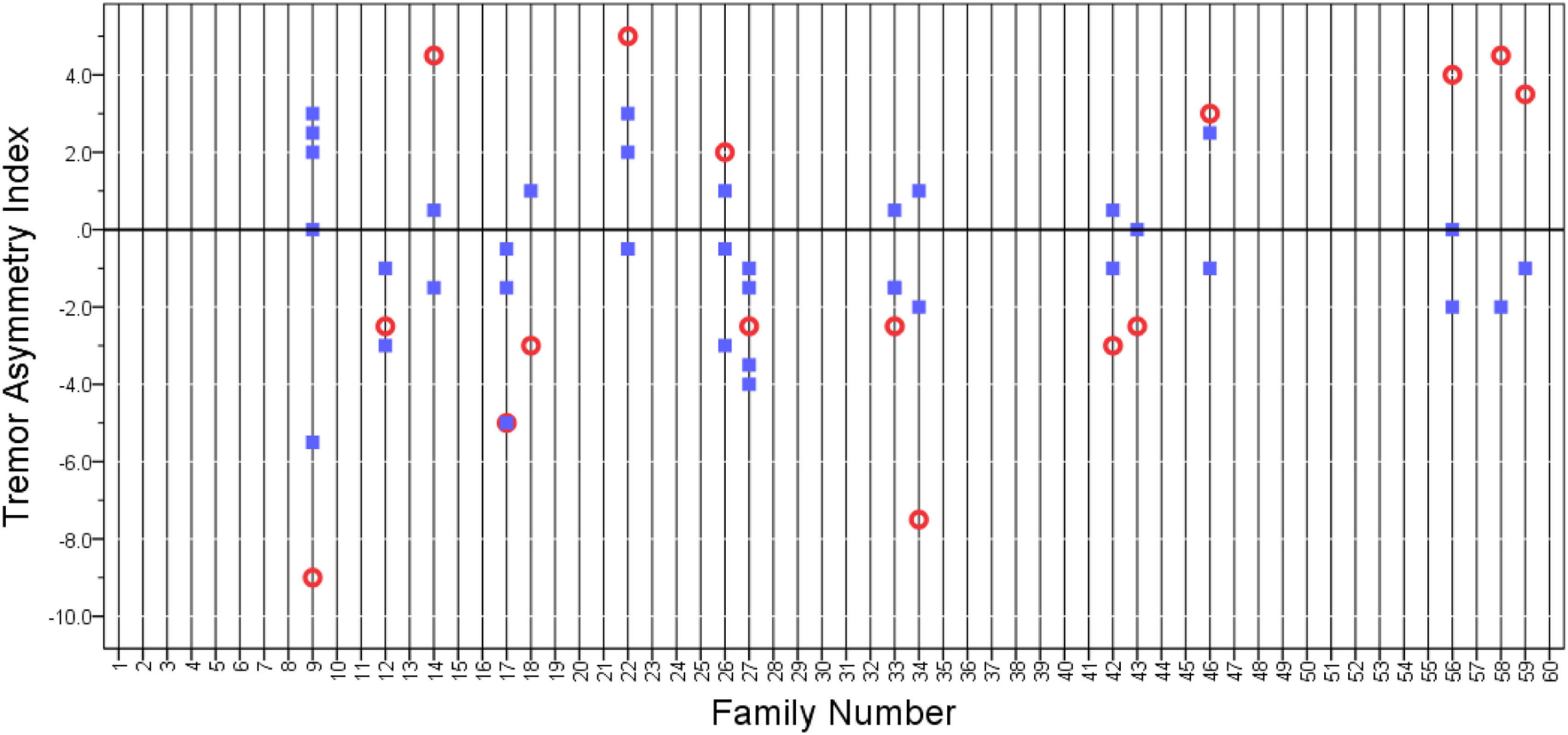
**Tremor asymmetry index in probands (open circles) and relatives (closed squares)**. We selected the extreme quartiles of probands whose tremor asymmetry index had extreme values. These were the 10% of probands whose tremor asymmetry index value was ≤−2.5 and the 10% of probands whose tremor asymmetry index value was ≥2.0. A value of 0 indicates that the tremor was equal on both sides. Positive values indicate that tremor is greater on the right side, and negative values indicate that tremor is greater on the left side. Vertical grid lines run through the data points in each family.

In a second additional analysis, we selected the probands whose tremor asymmetry index had extreme values (i.e., the top 10% of probands whose tremor asymmetry index value was ≤−2.5 and the bottom 10% of probands whose tremor asymmetry index value was ≥2.0). There were 16 such probands. We also included their 38 relatives in this analysis. There were rare families in which the asymmetry index was similar (e.g., Family 27 in Figure 3); however, for the most part, there seemed to be no pattern relationship of the relatives’ asymmetry index to that of the probands’ (Figure 3), and in the bivariate linear regression model, the tremor asymmetry index in the proband was not a predictor of the tremor asymmetry index in the relatives (beta = 0.03, *p* = 0.71).

Because tremor was assessed with an ordinal clinical rating scale rather than with accelerometry, we performed a third additional analysis in which we switched our focus from the degree to which tremor was asymmetric and focused instead on the presence or absence of asymmetry. Thus, cases were classified into three categories: (1) higher tremor score on right than left, (2) higher tremor score on left than right, and (3) tremor scores on both sides that were equal. In this bivariate linear regression model, this classification of tremor asymmetry in the proband did not predict this classification of tremor asymmetry in the relatives (beta = 1.01, *p* = 0.89).

In a fourth additional analysis, we repeated our main analyses, restricting the sample to right-handed individuals, and the results did not differ: in a bivariate linear regression model, the tremor asymmetry index in the proband was not a predictor of the tremor asymmetry index in the relatives (beta = 0.03, *p* = 0.68).

Finally, in an ANOVA that utilized data from 187 enrollees, we tested for heterogeneity across families in the tremor asymmetry index; in this model, the dependent variable was the tremor asymmetry index in the relatives, and the group factor was the family number. In this analysis, we did not find significant evidence of heterogeneity in the tremor asymmetry index across families (ANOVA *F* = 0.96, *p* = 0.56) (i.e., a significant proportion of the total variance in the tremor asymmetry index was not explained by the family grouping).

## Discussion

Whether the tremor asymmetry profile (i.e., differential expression of tremor in each arm) is similar across family members with ET is not known. This is an elemental question, yet surprisingly, there are no published data. Underlying this clinical question is the corollary hypothesis that there may be selective vulnerability of underlying pathophysiological factors across family members within an ET family. The pathophysiology of ET has not been fully elucidated although compelling data link it to the cerebellum and cerebellar pathways (21–25). In the current study, family membership was not an important contributor/predictor of tremor asymmetry. Hence, as a corollary, familial factors may not influence the expression of underlying pathophysiological factors that could be contributing to tremor asymmetry. Similarly, in a study of patients with PD, asymmetric motor features occurred equally in familial and sporadic cases, suggesting that the distribution of the nigrostriatal lesion exists in patients with either form of PD regardless of apparent genetic influence (3).

In complex diseases, vulnerability of specific neuronal populations is probably determined by both genetic and non-genetic (e.g., environmental) factors. Examples of this can be found in the PD literature. For example, *parkin* and rotenone, two prominent genetic and environmental factors linked to PD, are thought to act in an opposing manner on the same molecular target in the cell, microtubules, whose destruction underlies the selective vulnerability of dopaminergic neurons (26). It is reasonable to question whether in ET, genetic factors could be contributing to the selective vulnerability of specific neuronal populations to injury. However, the current data did not provide a concrete example of this.

How will these new data allow us to counsel ET patients? ET patients frequently search for predictors of the course their disease will take and in that search often draw comparisons with other affected relatives. Some tremor features aggregate in families, providing predictive information, as is the case with rate of progression of tremor (14), while others do not [e.g., in the case of presence of cranial tremor (15)]. However, with regards to asymmetry profile of tremor, there seems to be no familial pattern, and ET cases should not look toward their relatives for predictive information. The data presented here will enable clinicians to base discussions about the features of disease and disease course on published data and will provide ET cases and families with further information about the predictability of specific disease features.

This study had limitations. Tremor was assessed with an ordinal clinical rating scale rather than with accelerometry. The latter would have provided more precise estimates of tremor severity and would have lent greater precision to our major outcome variable. To try to deal with this issue, we performed an analysis in which we switched our focus from the degree to which tremor was asymmetric and focused instead on the simpler question—the presence or absence of asymmetry. Thus, cases were classified into three categories: (1) higher tremor score on right than left, (2) higher tremor score on left than right, and (3) tremor scores on both sides that were equal. Even in these analyses, tremor asymmetry in the proband did not predict tremor asymmetry in the relatives (beta = 1.01, *p* = 0.89). Second, the mix of families that we studied may not be representative of all ET families. The study also had strengths. First, the question we ask has not been addressed before so that there are no available data other than our own. Second, ET cases were carefully phenotyped by a senior neurologist with a particular expertise in tremor disorders. Third, tremor was rated with a rating scale that is reliable and valid (18, 20, 27). Fourth, the sample size was large, with data from more than 50 ET families. Fifth, we were able to examine a broad range of demographic and disease-linked factors that could have contributed to asymmetry. Finally, the data generated will provide added value to the clinical dialog, giving patients one more piece of information about the way the disease manifests within families.

## Ethics Statement

The study was approved by Columbia and Yale University Institutional Review Boards; participants signed written informed consent.

## Author Contributions

EL: research project conception, research project organization, research project execution, statistical analysis design, statistical analysis execution, statistical analysis review and critique, and manuscript preparation in writing of the first draft. NH, KC, KN, and JP: research project organization, research project execution, statistical analysis review and critique, and manuscript preparation with respect to review and critique. LC and RO: research project conception, research project organization, research project execution, statistical analysis review and critique, and manuscript preparation with respect to review and critique.

## Conflict of Interest Statement

The authors declare that the research was conducted in the absence of any commercial or financial relationships that could be construed as a potential conflict of interest.
